# Circulating tumor cells in neuroblastoma: Current status and future perspectives

**DOI:** 10.1002/cam4.4893

**Published:** 2022-05-27

**Authors:** Ran Yang, Shan Zheng, Rui Dong

**Affiliations:** ^1^ Department of Pediatric Surgery Children's Hospital of Fudan University Shanghai China

**Keywords:** circulating tumor cell, disseminated tumor cell, liquid biopsy, minimal residual disease, neuroblastoma

## Abstract

Neuroblastoma is the most common extracranial solid tumor in children, accounting for 10% to 20% of deaths of pediatric malignancies. Due to the poor prognosis and significant biological heterogeneity of neuroblastoma, it is essential to develop personalized therapeutics and monitor treatment response. Circulating tumor cells (CTCs), as one of the important analytes for liquid biopsy, could facilitate response assessment and outcome prediction for patients in a non‐invasive way. Several methods and platforms have been used for the enrichment and detection of CTCs. The enumeration of CTCs counts and evaluation of tumor‐specific mRNA transcript levels could provide prognostic information at diagnosis, during or after chemotherapy, and during the process of disease progression. So far, studies into neuroblastoma CTCs are only in the preliminary stages. The quality‐controlled large prospective cohort studies are needed to evaluate the clinical significance and statistical rigor of CTC detection methods. Moreover, there remains a lot to be explored and investigated in genotyping characterization of neuroblastoma (NB) CTCs and construction of in‐vitro or in‐vivo functional models. CTCs and circulating tumor DNA (ctDNA) analysis will be complementary in understanding tumor heterogeneity and evolution over the course of therapy for patients with NB in the future.

## Graphical abstract

In this review, we summarized current status of research on circulating tumor cells (CTCs) in pediatric neuroblastoma, including enrichment/detection methods and the prognostic value of CTCs in clinical practice. So far, studies into neuroblastoma CTC are only in the preliminary stages. There remains a lot to be explored concerning CTC transcriptional and genome alterations, functional properties and clinical utility for personalized drug screenings.

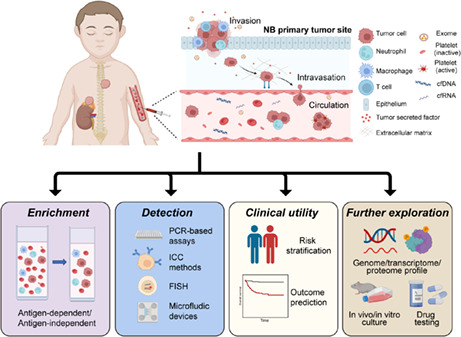

## 1 INTRODUCTION

As the fourth most common pediatric malignancy and the most common extracranial solid tumor during childhood, neuroblastoma (NB) occurs in approximately 25–50 cases per million individuals and accounts for 10% to 20% of deaths of pediatric malignancies.^1^ NB is surmised to arise from developing neural crest cells, including the sympathoadrenal lineage of neural crest cells (NCCs) and Schwann cell precursors, thus affecting paraspinal ganglia as well as adrenal medulla.^2^ According to risk classification criteria developed by the International Neuroblastoma Risk Group (INRG), NB can be categorized into very low‐risk, low‐risk, intermediate‐risk, and high‐risk groups based on the assessment of age at diagnosis, INRG tumor stage, histologic category, grade of differentiation, DNA ploidy, *MYCN* status, as well as copy number status at chromosome *11q*. The 5‐year event‐free survival (EFS) for low‐risk group patients is over 75%, while it is less than 50% for the high‐risk group.^3^

Due to the poor prognosis and significant biological heterogeneity of NB, it is essential to develop personalized therapeutics and monitor treatment response. Invasive measurements, including tumor biopsy, bone marrow (BM) biopsies, and aspirates are required for diagnosis and evaluation of metastasis, which are also fundamental for risk classification, assignment of therapeutic strategies, and evaluation of treatment effects. However, these procedures are invasive and painful for patients. Moreover, non‐invasive tests such as serial computed tomography (CT) scan, and evaluation of metabolites including urinary vanilly‐mandelic acid and homovanillic acid, serum lactate dehydrogenase, and neuron‐specific enolase (NSE) are not sensitive and specific enough to monitor relapse or therapeutic response.^4^ Therefore, it is critical to utilize a new method to detect early NB metastasis lesions, evaluate treatment effects, and monitor tumor recurrence.

The past few years have seen the emergence and rapid development of a novel diagnostic concept known as “liquid biopsy.”^5^, ^6^ Liquid biopsy allows for the collection and analysis of tumor‐derived circulating biomarkers, including circulating tumor cells (CTCs), extracellular vesicles, tumor‐educated platelets, and cell‐free DNA (cfDNA) or RNA (cfRNA),^7^ from peripheral blood (PB) or body fluids such as saliva and urine.^8^ Compared with conventional diagnostic techniques, liquid biopsy is an appealing non‐invasive approach, which offers an opportunity for real‐time tracking of disease progress and longitudinal evaluation of therapeutic effects.^9^

As vital components for liquid biopsy, CTCs refer to malignant cells emigrating from primary tumor lesions and circulating in the blood.^10^ CTCs were described for the first time in 1869 when Thomas Ashworth found cells in the blood resembling those in the tumor at autopsy.^11^ In terms of ways into blood vessels, it is believed that CTCs can either initially enter the circulation through phenotype change and interaction with tumor microenvironment cells or passively infiltrate into the bloodstream through leaky and tortuous vasculature in the tumor, resulting in a rather heterogeneous cell group.^12^, ^13^, ^14^ Most CTCs died in circulation out of fluid shear stresses, anoikis or immune pressures. A fraction of CTCs is able to survive and persist in distant sites (e.g., BM),^15^, ^16^ which is called disseminated tumor cells (DTCs). There are two reported possible mechanisms involved in the dissemination of CTCs. The most well‐recognized one is the epithelial‐mesenchymal transition (EMT), through which CTCs acquire a more motile phenotype. Apart from EMT, mesenchymal‐amoeboid‐transition has been recently discovered in several tumors, including melanoma and small‐cell lung cancer.^17^, ^18^ CTCs can either travel alone as single cells or aggregate as cell clusters. Although rarer than CTC single cells, CTC tumor clusters have estimated 20–100 increased metastatic potential and are associated with a more aggressive phenotype, as revealed in breast and prostate cancer.^19^, ^20^, ^21^, ^22^ Most recently, non‐malignant cells such as neutrophils and cancer‐associated fibroblasts have been found to cluster with CTCs and facilitate metastasis seeding in lung, breast, and prostate cancer.^23^, ^24^, ^25^, ^26^ The biology of CTCs and their interaction with other cell types in the bloodstream is a complicated field that might shed lights on the onset and progression of metastasis and needs further exploration.

Compared with cell‐free nucleic acids and extracellular vesicles, CTCs are intact tumor cells released from original or metastatic lesions, capable of providing abundant and comprehensive information on tumor on DNA, RNA, protein, and metabolites levels. As estimated by mouse models and CTC enumeration of breast cancer patients immediately after removal of primary tumors, the median half‐life of a single CTC is very short, measured in hours, while it is even shorter for CTC clusters.^20^, ^27^, ^28^, ^29^ This transient feature enables CTCs to become a promising dynamic marker revealing tumor progression and evolution.

As one of the highly aggressive tumors, NB has been demonstrated to frequently shed tumor cells into circulation at diagnosis or after surgery and chemotherapy. The first study that identified NB CTCs was in 1979 when NB cell culture was established from PB from a patient with disseminated diseases.^30^, ^31^ It was revealed that CTCs could be detected in about 70% of high‐risk NB patients and around 50% of stem cell harvests.^32^, ^33^, ^34^ CTC clusters were also observed in high‐risk NB.^10^, ^35^ Herein, in this review, we will focus on the current status of the detection and collection methods for NB CTCs. The prognostic roles of CTCs in predicting tumor risk‐stratification and overall survival (OS) reported in the previous studies are also reviewed. Moreover, we will further discuss the difficulties of the application of CTCs in NB for now and its future perspectives.

## 2 ENRICHMENT AND DETECTION METHODS FOR CTCS IN NB

### 2.1 Enrichment

**FIGURE 1:**
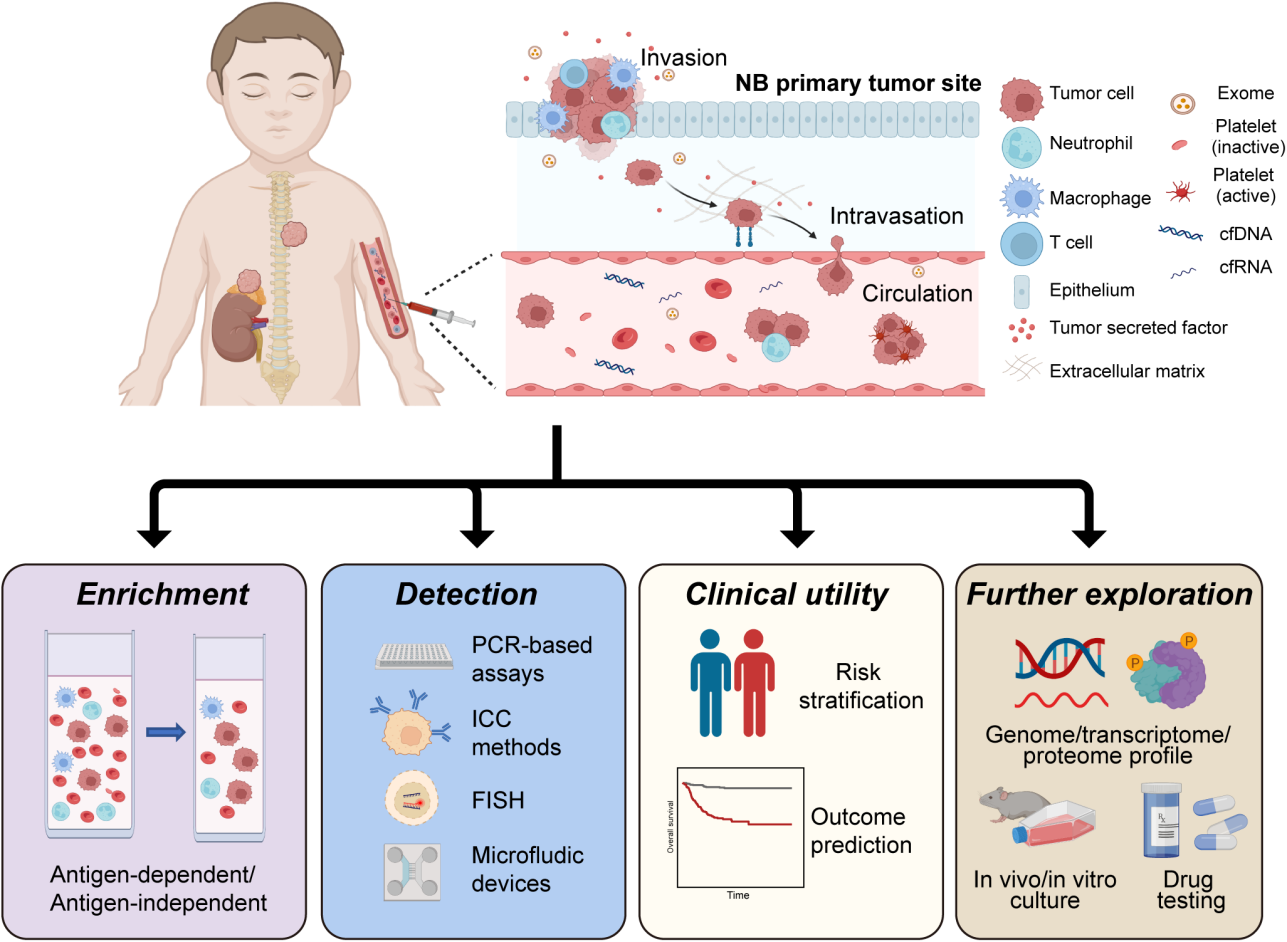
Overview of identification and application of circulating tumor cells in neuroblastoma. cfDNA, cell‐free DNA; cfRNA, cell‐free RNA; FISH, fluorescence in situ hybridization; ICC, immunocytochemical; NB, neuroblastoma; PCR, quantitative polymerase chain reaction (Created with BioRender.com).

The number of CTCs in PB is meager, counting up to several or a few hundred per milliliter. Therefore, the process of enrichment of CTCs is usually necessary. Generally, there are two main categories of CTC enrichment and detection techniques: antigen‐independent or antigen‐dependent methods (Figure 1).

Antigen‐independent methods are based on differences in physical properties between CTCs and non‐malignant blood cells such as cell size, density, electric charges, and deformability. One study has developed acoustophoresis, a label‐free microfluidic technology utilizing ultrasound waves in microchannels, to separate NB tumor cells from peripheral blood stem cells (PBSC) based on different cell size distribution between malignant and normal cells.^36^ However, this method was limited by low throughput with 10^5^ cells/min and limited purity out of heterogeneity in NB tumor cell size.

Antigen‐dependent approaches rely on differential expression of cell‐surface markers, including positive selection and negative selection. CTCs in NB are enriched by fluorescence‐activated cell sorting (FACS) and magnetic cell selection either through positive selection using tumor‐specific markers, i.e., anti‐GD2 or anti‐CD56 antibodies^37^ or through negative selection using anti‐CD45 antibodies to remove cells of hematopoietic origin^10^, ^38^ (Table 1).

**TABLE 1:** Summary of enrichment and detection methods used in NB CTCs

Process	Methods	Application	Advantage	Disadvantage	Reference
Enrichment	Antigen‐independent: acoustophoresis	A microfluidic technology using standing ultrasound waves to identify cells with different sized distributions	Independent of cell labelling Little effect on cell viability	Low specificity	^36^
	Antigen‐dependent: FACS, magnetic cell selection	Negative selection using anti‐CD45 antibodies to remove cells of hematopoietic origin or through positive selection using tumor‐specific markers, i.e., anti‐GD2 or anti‐CD56 antibodies	High specificity	Reliance on specific antibodies	10, 36
Detection	ICC methods	Detection of CTCs using antibodies targeting NB‐specific markers including GD2, CD56 and CD90 Allowing for the identification of around 1–2 CTCs among 10^5^ PBMCs	High specificity Golden standard	Low sensitivity Reliance on specific antibodies	41, 42, 43, 44
	PCR‐based methods (RT‐PCR, ddPCR)	Evaluation of either single or panels of tumor mRNA transcripts level in the blood (*TH*, *PGP9.5*, *GALGT*, *PHOX2B*, *DDC*, *DCX*, *DBH*, *ELAVL4*, *CRAMP1*, *GAP43*, *ISL1*, *CHRNA3*, *KIF1A*); Allowing for detecting 1 CTC in 10^3^ PBMCs to 10^7^ PBMCs.	High sensitivity	Low specificity Inconsistency among studies	44, 49, 53, 56, 57
	FISH	Liu et al. used immunostaining of CD45 and DAPI, and FISH of the CEP8 to detect CTCs. Cells with DAPI+/CD45‐/CEP8 ≥3 were defined as NB CTCs	High sensitivity	Reliance on specific hyperdiploidy of chromosomes in tumor cells	^10^
	Amnis Image Stream Imaging Flow Cytometer (ISx)	A high‐throughput method combines the features of FACS with fluorescence microscopy allowing for the identification GD2+/CD45‐ CTCs in a short period	High throughput	Missing a small proportion of NB tumor cells without GD2 expression	^58^
	DEPArray	A high‐throughput method utilizes dielectrophoresis to electronically trap and move fluorescence‐labeled (GD2, CD45, CD56) individual cells	High throughput	Missing a small proportion of NB tumor cells without GD2 expression	^59^

Abbreviations: ddPCR, droplet digital PCR; FISH, fluorescence in situ hybridization; ICC, immunocytochemical; NB, neuroblastoma; RT‐PCR, reverse transcription quantitative polymerase chain reaction.

Recently, combined applications of different techniques have been developed, enabling the acquisition of CTCs with higher purity and recovery rate. CTC‐iChip and GEDI‐Chip, which are both integrated microfluidic CTC capture platforms, are capable of sorting CTCs from PB with high purity in breast and prostate cancers, based on the combination of multiple properties including cell size‐based separation and immuno‐magnetic beads selection.^39^, ^40^ These approaches hold a promising future in the application of NB CTC enrichment.

### 2.2 Detection and enumeration

Followed by enrichment, CTCs can be detected and enumerated by immunocytochemical (ICC) methods, FACS, reverse transcription quantitative polymerase chain reaction (RT‐qPCR), quantitative reverse transcriptase‐polymerase chain reaction (QRT‐PCR), droplet digital PCR (ddPCR), and fluorescence in situ hybridization (FISH) (Table 1) (Figure 1).

Immunocytochemical methods have been applied to detect NB CTCs using monoclonal antibodies targeting NB‐specific neuron markers including GD2, CD56 and CD90 (THY1).^41^, ^42^, ^43^ ICC allows for the identification of around 1–2 CTCs among 10^5^ peripheral blood mononuclear cells (PBMCs)^44^ without previous enrichment. Though the reliability of CTC detection and qualification in NB by ICC methods has remained inconclusive, it is recommended by INRG to use GD2 for the detection of rare NB tumor cells in BM, PB and PBSC through ICC.^37^

Compared with ICC methods, RT‐PCR or ddPCR have been more widely used to detect low‐abundance tumor mRNA transcripts in blood, enabling the indirect qualification of NB CTCs. The expression level of multiple NB‐specific neuron genes has become surrogates for NB CTC enumeration, including tyrosine hydroxylase (*TH*),^45^, ^46^ ubiquitin C‐terminal hydrolase L1 (*UCHL1/PGP9.5*),^47^ GD2 synthetase (*GALGT*),^48^ paired like homeobox 2B (*PHOX2B*),^48^ dopa decarboxylase (*DDC*), doublecortin(*DCX*), dopamine beta‐hydroxylase (*DBH*),^49^ ELAV like RNA binding protein 4 (*ELAVL4*),^50^ cramped chromatic regulator homolog 1 (*CRAMP1*), growth associated protein 43 (*GAP43*) and ISL LIM Homebox1 (*ISL1*).^51^ Among all utilized genes, *TH* is the most widely used one, whereas *PHOX2B* has shown to be the most sensitive and specific but limited by heterogeneous expression in tumors.^52^

Out of different sensitivity and variant gene expressions in tumor cells, the combination of multiple genes may have superiority in detecting CTCs.^53^ By comparing the expression profile of NB tumor and normal PB or BM samples through genome‐wide expression microarray analysis or serial analysis of gene expression libraries, various sets of markers have been selected to detect CTCs/DTCs such as six gene sets in BM (*CCND1*, *DDC*, *GABRB3*, *ISL1*, *KIF1A*, *PHOX2B*),^54^ and three markers for PB sample (*DCX*, *PHOX2B*, *TH*).^55^ One study also identified different panels of genes for PB and BM samples. This includes *PHOX2B*, *TH*, *DDC*, *DBH*, and *CHRNA3* for PB, while *PHOX2B*, *TH*, *DDC*, *CHRNA3*, and *GAP43* for BM samples.^53^ Panels of five NB‐mRNAs (*CHGA*, *DCX*, *DDC*, *PHOX2B*, and *TH*)^49^ and seven NB‐mRNAs (*CRMP1*, *DBH*, *DDC*, *GAP43*, *ISL1*, *PHOX2B*, and *TH*)^51^ have also been developed to detect CTCs both in PB and BM for high‐risk NB patients.

The sensitivity of PCR‐based analysis varied between studies, ranging from detecting 1 CTC in 10^3^ PBMCs to 10^7^ PBMCs.^44^, ^49^, ^53^, ^56^, ^57^ This difference is partially due to the application of different PCR‐based methods (e.g., RT‐PCR, QRT‐PCR, ddPCR) as well as the selection of distinct target mRNAs. So far, TH was the only mRNA recommended by INRG for detecting tumor cells in BM, PB, or PBSC by QRT‐PCR.^37^

Besides IC and RT‐qPCR, Liu et al. performed a negative selection of tumor cells using CD45‐coated magnetic beads to deplete non‐tumor cells. CTCs were further identified by FISH of the centromere of chromosome 8 probe (CEP8) and immunostaining of CD45, DAPI. Cells with DAPI+/CD45‐/CEP8 ≥3 were defined as NB CTCs. This method not only found single CTCs but also identified NB CTC clusters from a high‐risk patients.^10^

ICC, RT‐PCR, and FISH are confined to low throughput. Merugu et al. firstly used the Amnis Image Stream Imaging Flow Cytometer (ISx) to enumerate GD2+/CD45‐ NB CTCs from blood and DTCs from BM which enables analysis of large numbers of cells in a short time, and reported 0–264 NB CTCs/ml with a mean of 12 CTCs/ml at diagnosis for high‐risk NB patients.^58^ Another microfluidic and dielectrophoretic approach‐DEPArray which utilized dielectrophoresis to trap and move single cells, has also been adopted to capture individual NB tumor cells from GD2/CD45 labeled BM samples with the sensitivity of detecting one tumor cell per 10^6^ white blood cells.^59^ Both methods are performed in a high‐throughput manner with a high speed of sample processing. However, they might miss a small proportion of tumor cells without GD2 expression. Further studies should compare the sensitivity of the ISx and DEPArray with other methods (e.g., ICC, PCR‐based assays, FISH).

## 3 CLINICAL UTILITY OF CTCS IN NB

### 3.1 Prognostic value of CTCs

CTCs enumeration and tumor‐specific mRNA have been revealed to be valuable in the evaluation of disease progression and metastasis. Detection of CTCs and DTCs in PB or BM at diagnosis, during or after treatment, and in the relapsed tumor are significantly correlated with systemic metastases and low survival rate in NB patients^10^, ^38^, ^45^, ^46^, ^51^, ^54^, ^60^, ^61^ (Figure 1).

CTCs enumeration and transcript levels at diagnosis could be utilized to predict the probability of metastasis or tumor occurrence, response to chemotherapy, as well as patient survival. CTC counts were positively correlated with NSE levels while no significant correlation was found between CTC counts and *MYCN* status. Patients with ≥3 CTCs per 4 ml of PB at diagnosis are more likely to have metastasis than those with fewer CTCs (sensitivity, 88.89%; specificity, 78.59%).^10^ Patients with a cut‐off value ≥10 CTCs/4 ml in PB tended to have lower OS with a hazard ratio (HR) of 6.279 based on univariate Cox regression analysis. Furthermore, multivariable Cox regression analysis including NSE levels, INSS stage, and CTC counts showed that the cut‐off value was not an independent risk factor.^10^ In the meantime, in another cohort study of 210 NB patients, positive *TH* expression in PB at diagnosis was an independent poor prognostic factor for progression‐free survival (HR, 6.35).^45^ Detection of tumor‐specific mRNA expression such as *TH* and *DCX* in PB from patients with localized NB at diagnosis was associated with a higher probability of relapse and poorer survival.^60^ Even among high‐risk groups, high *TH* or *PHOX2B* expression (log10 TH >0.8, log10 PHOX2B >0.28)^61^ or the presence of ≥100 tumor cells per 10^5^ nucleated cells in BM at diagnosis^33^ were correlated with even poorer outcomes. Therefore, patients with a high level of *TH* and *PHOX2B* at diagnosis were defined as ultrahigh‐risk by European HR‐NBL1/SIOPEN study, requiring novel treatment strategies.^61^ Moreover, higher CTC counts in PB at diagnosis were positively correlated with an incomplete BM response after induction therapy and disease progression after treatment.^10^, ^58^

Quantifying tumor cells in PB or BM during induction therapy or in the remission stage after completion of treatment may provide insights into minimal residual disease (MRD) and tumor occurrence when data from a conventional evaluation of relapse or metastasis are ambiguous. Since MRD are drug‐resistant tumor cells that persist after initial seemingly successful treatment, it may lead to tumor occurrence after abundant chemotherapy and correlated with inferior survival.

Evidence has shown that a high level of *TH* mRNA in PB and *DCX* in BM signified poorer EFS and OS when detected after induction chemotherapy and at the end of treatment.^46^ Based on transcriptome‐wide profiling, eight markers (*CCND1*, *CRMP1*, *DDC*, *GABRB3*, *ISL1*, *KIF1A*, *PHOX2B*, and *TACC2*) were found abundantly expressed in NB tumor tissues Stage 4 NB patients while no detection in normal marrow or blood samples, which could be utilized in MRD detection.^54^ Among eight markers, expression of *CCND1*, *DDC*, *GABRB3*, *ISL1*, *KIF1A*, and *PHOX2B* in 116 marrows after two treatment cycles was a prognostic of worse PFS and OS by Kaplan–Meier analyses. Another study revealed that the expression of seven NB mRNAs (*CRMP1*, *DBH*, *DDC*, *GAP43*, *ISL1*, *PHOX2B*, and *TH*) in PB and BM varied with disease status and collection time point, the levels of which in post‐treatment BM had prognostic value in tumor relapse.^51^ Apart from tumor‐specific mRNAs, CTCs counts are also of great significance in evaluating MRD. There is a strong correlation between relapse‐free survival and the absence of cell surface vimentin (CSV) + NB CTCs for NB patients under remission, as shown in a Phase II long‐term preventive clinical trial of 93 patients. Compared with >3+ CSV^+^ CTCs, none or only 1–2 CSV^+^/6 ml CTCs were deemed as low tumor burden and yielded a faster reduction of relapse risk.^38^

Furthermore, in relapsed or refractory NB patients, the expression level of NB‐specific mRNA in BM or PB samples can quantify disease burden and predict patient survival. Marachelian et al. found in a prospective study of 101 patients with relapsed/ refractory NB that higher levels of five NB‐specific mRNA (*CHGA*, *DCX*, *DDC*, *DCX*, *PHOX2B*) in NB were significantly correlated with progression‐free survival independent of clinical disease and MYCN status.^49^

To a certain extent, these results have revealed the significance of monitoring the dynamic change of CTCs counts or tumor‐specific mRNA levels throughout the whole disease course, which could facilitate risk classification and outcome prediction. However, current assays for NB CTC detection utilize different NB‐associated antigens or mRNAs. We will require the quality‐controlled large prospective cohort studies to evaluate the clinical significance and statistical rigor of these assays.

### 3.2 The significance of CTCs detection in autologous stem cell transplantation

High‐risk NB patients often receive myeloablative chemotherapy with the rescue with autologous stem cell transplant. Several studies have found contamination of CTCs in PBSC with various frequencies ranging from 9% to 58%.^32^, ^62^, ^63^ It remains obscure whether reinfused tumor cells in autologous grafts could contribute to relapse after transplantation in NB patients.

Chambon et al. found that patients positive for TH mRNA assessed by RT‐PCR in PBSC had a lower 2‐year post‐harvest survival than those with negative TH mRNA.^64^ Another study used retrovirally mediated gene marking techniques to BM harvests from eight NB patients and found that genetically‐labeled tumor cells in non‐purged BM harvests contributed to relapse after myeloablative therapy in 3 patients.^65^

However, there are also research revealing that graft contamination was not significantly related to an unfavorable outcome. Several small cohort studies showed that patient survival was not affected by PBSC contamination in stage IV or high‐risk NB, using a different combination of markers including *TH* and *GD2S* ^66^ or *TH* alone.^63^ In a retrospective cohort of 104 high‐risk NB patients without MRD in BM, the presence of tumor cells in PBSCs was estimated by five NB‐specific markers (*PHOX2B*, *TH*, *DDC*, *CHRNA3*, and *DBH*) was not indicated a worse survival in univariate and multivariate analysis.^62^

The differences in the results may be because the selection of NB‐associated mRNAs varied between studies. However, it has been shown that though at a lower level, *TH* and *B4GALNT1* mRNA can also be detected in healthy BM, PB, and PB stem cells.^67^, ^68^, ^69^ Reliance on only one or two tumor‐associated mRNAs could lead to the overestimation of graft contamination.

Though the association between PBSC contamination and patient outcome was controversial, non‐purged PBSCs are generally acceptable as support for myeloablative therapy of high‐risk NB. In a large randomized Phase 3 clinical trial of Children's Cancer Group enrolling 495 patients, immunomagnetic purging of PBSC using five monoclonal antibodies targeting cell surface antigens (126‐4, 390, 459, HSAN1·2, and BW575) had no effect on improving outcome. Little effect of purging of PBSC might be due to incomplete elimination of tumor cells or residual tumor in patients.^32^

## 4 CTCS VERSUS ctDNA IN THE CLINICAL SETTING

Although many methods and technologies have been developed over the past decades to enrich and detect CTCs from NB patients, this work remains challenging.

In light of recent advances in next‐generation sequencing and DNA extraction methods, the analysis of circulating tumor DNA (ctDNA), a fraction of cfDNA and representative of intra‐tumoral heterogeneity, can be helpful in monitoring clonal evolution and treatment response.

NB has been revealed to shed large amounts of ctDNA in the bloodstream compared with other pediatric cancers,^70^ with a high cfDNA level and a high ratio of ctDNA in the cfDNA at diagnosis.^71^ cfDNA concentration might be a promising prognostic biomarker for NB. Elevated total cfDNA levels in the plasma indicated a heavier tumor burden, associated with tumor staging, *MYCN* amplification (MNA), primary tumor sites, and tumor metastasis.^72^, ^73^ ctDNA concentration in plasma or serum from patients with NB can be used for monitoring clinical response and detecting early tumor occurrence.^74^, ^75^, ^76^ Apart from cfDNA level, recent studies have also demonstrated the detection of various NB‐specific genetic and epigenetic alterations in cfDNA from patients during the course of disease, including MNA,^71^, ^77^, ^78^, ^79^ *ALK* amplification or mutation,^78^, ^80^ segmental chromosome alterations (17q gain, 11p loss),^80^, ^81^, ^82^ and epigenetic modifications (hypermethylation of *RASSF1A* and *DCR2*, 5‐hydroxymethylcytosine profiles).^74^, ^83^, ^84^, ^85^ In addition to the identification of canonical NB‐specific genome alterations by PCR‐based methods (qPCR, ddPCR), comprehensive genomic analysis methods including targeted panel sequencing of cancer‐associated genes, whole‐exome sequencing, and whole‐genome sequencing allow detailed characterization of patient‐specific ctDNAs and provide insights into its prognostic and surveillant utilities.^70^, ^71^, ^86^, ^87^ Analytical targets used in NB ctDNA studies and a comparison of various methods for detecting genomic aberrations were summarized in Tables 2 and 3, respectively.

**TABLE 2:** Summary of commonly‐used analytical targets used in NB ctDNA studies

Target	Sample type	Method	Clinical utility	Reference
*MYCN* amplification	Plasma, serum	qPCR, ddPCR, WES, WGS	Predicting *MYCN* status of tumor tissue and reflecting intra‐tumoral heterogeneity Risk stratification and therapeutic decision‐making Monitoring treatment response and reflecting on early relapse	71, 75, 77, 78, 103, 104
*ALK* amplification/mutations	Plasma, serum	qPCR, ddPCR, WES, WGS	Facilitating detection of druggable *ALK* alterations (p.F1174L, p.F1245I, p.R1275Q) Risk stratification and therapeutic decision‐making Monitoring treatment response and reflecting on early relapse	73, 75, 78, 80
17q gain	Plasma, serum	qPCR, SNP array OncoScan array, WES	Predicting chromosomal alterations of tumor tissue Risk stratification	71, 82, 105
11q loss	Plasma, serum	SNP array, OncoScan array, WES, microsatellite analysis	Predicting chromosomal alterations of tumor tissue Risk stratification	71, 81, 105
*RASSF1A* hypermethylation	Plasma, serum	Methylation‐specific PCR	Predicting poor outcome Monitoring treatment response and reflecting on early relapse	74, 85
*DCR2* hypermethylation	Plasma, serum	Methylation‐specific PCR	Predicting poor outcome Monitoring treatment response and reflecting on early relapse	84, 106
5‐hydroxymethylcytosine profiles	Plasma	Nano‐hmC‐Seal technology	Indicating metastatic burden Detecting potential biomarkers for treatment response and outcome prediction	^83^
Comprehensive genome analysis of cfDNA	Plasma	WES, WGS	Alternative for somatic tumor mutation and copy‐number profiling Reflecting spatial and temporal heterogeneity Demonstration of clonal evolution during follow‐up and identification of treatment‐resistant clones	70, 71, 86, 87
Targeted sequencing panel of cancer‐associated genes	Plasma	Targeted sequencing	Designing NB driver genes panels used for cfDNA analysis Identify potential pathogenic variations used for clinical and pathological diagnosis	^107^

Abbreviations: cfDNA, cell‐free DNA; ddPCR, droplet digital PCR; NB, neuroblastoma; qPCR, quantitative PCR; SNP array, single nucleotide polymorphism array; WES, whole‐exome sequencing; WGS, whole‐genome sequencing.

**TABLE 3:** Summary of ctDNA profiling methods used in NB

Methods	Advantage	Disadvantage	Reference
PCR‐based methods (qPCR, ddPCR, methylation‐specific PCR)	Rapid and cost‐effective identification of tumor‐specific genomic alterations in cfDNA	Rely on tumor‐specific genomic alterations Prone to technical bias	74, 75, 77, 78, 80, 84, 85, 103, 104
Chromosomal micro‐array analysis (SNP array, CGH array, OncoScan array)	Identification of patient‐specific tumor genomic copy number profiles and intra‐tumoral heterogeneity	Lower sensitivity and false positives due to the masking effect of healthy cfDNA Mainly provide CNV information	78, 105
WES/WGS	Comprehensive detection of patient‐specific tumor genomic alterations and therapy‐resistant clones	Detection of large numbers of variants with uncertain significance Expensive and time‐consuming Difficulties in data interpretation	70, 71, 86, 87
Targeted panel sequencing	Cost‐effective identification of patient‐specific driver or druggable mutations	Limited capability in detecting large chromosomal aberrations Rely on optimal panels of cancer‐associated genes	^107^
5‐hydroxymethylcytosine profiles sequencing (Nano‐hmC‐Seal)	Independent of somatic mutations and not restricted by low mutational burdens	Require the validation of large‐cohort prospective studies	^83^

Abbreviations: cfDNA, cell‐free DNA; CGH array, comparative genomic hybridization array; CNV, copy number variation; ddPCR, droplet digital PCR; qPCR, quantitative PCR; SNP array, single nucleotide polymorphism array; WES, whole‐exome sequencing; WGS, whole‐genome sequencing.

Generally, as one of the important analytes in liquid biopsy apart from CTC, ctDNA also provides a promising, highly sensitive, and cost‐efficient tool for monitoring disease status and treatment responsiveness in patients with NB. Compared with CTC enrichment and detection technologies, methods for ctDNA identification and qualification are more cost‐efficient and straightforward. Moreover, it also requires smaller sampling volumes of biofluids, which facilitates longitudinal sampling during the disease course. One proof‐of‐concept study reported the feasibility of monitoring NB‐specific genomic alterations from ctDNA in minimal volumes of biofluids from infants (recommendations: 1 ml of blood and BM plasma, 2 ml of cerebrospinal fluid).^88^ However, the clinical application of ctDNA analysis also has its inevitable drawbacks. First, ctDNA is thought to originate from lytic, apoptotic or necrotic tumor cells. Therefore, some genomic alterations detected in ctDNA may not be relevant to disease progression and warrant more careful inspection. Second, ctDNA only carries genomic information of malignant cells while CTCs can provide comprehensive tumor profiles, including DNA, RNA, proteins, and metabolites signatures. Finally, in vivo or in vitro drug sensitivity testing requires living tumor cells which need ctDNA analysis could not satisfy. Collectively, both CTC and ctDNA analysis have their strengths and weaknesses. It's likely that these two approaches will be complementary in providing information on tumor heterogeneity and helping clinical decision‐making for patients with NB, as suggested in prostate and breast cancer studies.^89^, ^90^

## 5 FUTURE PERSPECTIVES AND CHALLENGES OF CTCS UTILIZATION IN NB

Though an increasing number of small cohort studies have attempted to investigate the clinical and prognostic value of CTCs in NB, no individual assays have been validated by large cohort prospective studies and approved for routine clinical use. Current CTC studies in NB are only confined to CTC detection and enumeration, indicating that this field in pediatric oncology lags behind its adult counterparts. There remains a lot to be explored concerning CTC transcriptional and genome alterations, functional properties and clinical utility for personalized drug screenings (Figure 1).

### 5.1 The full exploration of information embedded in CTCs

In NB, CTC counts or several tumor‐specific transcripts have been analyzed and correlated with clinical prognosis. Few studies have investigated the genome, transcriptome, or proteome profile of CTCs.^91^ Genotypic characterization of CTCs could provide insights into NB metastasis and initiation mechanisms, as well as tumor evolution along the disease course.

Comparison of genome aberrations in CTCs with primary and metastasis lesions can reveal chromosomal aberrations or copy number alterations (CNA) throughout the process of metastasis and tumor cell homing. Single‐cell sequencing analysis on several tumors has confirmed CNA convergence from the primary tumor to CTCs.^92^

Compared with adult cancer types, NB in children displayed a low somatic mutation burden with few recurrent single nucleotide variants, short insertions, and deletions(indels),^93^ which indicates that epigenetic modifications and transcriptome changes might be of great significance. Single‐cell RNA sequencing and ATAC‐sequencing are two advanced and powerful technologies that could be applied to analyze transcriptomic shift and heterogenous phenotype of CTCs, unraveling specific gene regulatory networks and molecular pathways.^94^ Moreover, the development of techniques for CTC proteomic analysis enables dynamically identifying heterogeneous expression of CTC proteins and cancer cells potentially responsible for treatment resistance.^95^, ^96^, ^97^

### 5.2 Functional assay: Ex vivo CTC culture and CTC‐derived xenograft models

Functional properties of CTCs in NB can be further explored through ex‐vivo culture and CTC‐derived xenograft (CDX) models.

Ex vivo culture of CTCs could be utilized to conduct additional functional assays, as indicated in breast cancer that isolated CTCs lines provide abundant resources for testing drug sensitivity.^98^ Persistent CTC‐derived cell lines have been established in breast and colon cancer.^98^, ^99^ These cell lines have shown specific signatures, including stemness, DNA‐repair phenotype as well as a high metabolic rate, when compared with primary tumor cells.

CDX models could faithfully recapitulate molecular and cellular features of CTCs, and reveal properties required for metastasis and tumor resistance. CDXs have recognized CTC populations expressing *EPCAM*, *CD44*, *CD47*, and *MET* as metastasis‐initiating cells in breast cancer.^100^ Comparison of the transcriptome of chemo‐sensitive and chemo‐resistant CDX models confirmed increased intra‐tumoral heterogeneity after chemotherapy and suggested multiple concurrent resistant mechanisms in small‐cell lung cancer.^101^ In addition, since CDXs mimic patient tumor genomics and response to chemotherapy, these models could be utilized for drug screens and sensitivity testing.

Until now, few studies are reporting the construction of persistent CTC cell lines or CDXs derived from NB, leaving an interesting and exciting field to explore.

### 5.3 Challenges of clinical utility

Even though CTCs can add additional values in diagnosis and monitoring treatment response, currently, the unreliable and infrequent detection in NB patients impeded its clinical utilization. The first challenge is that NB‐specific cell surface markers have yet to be discovered. Widely‐used positive selection makers, disialoganglioside GD2, and adhesion molecule CD56 have limited sensitivity and specificity. GD2 is only expressed on a subset of NB tumor cells^59^ while CD56 is also expressed on a portion of natural killer cells.^102^ The second is that limited volumes of blood can be obtained from NB patients, whereas the in‐depth investigation of genotyping characterization and functional properties requires a relatively large number of CTCs. The suggested volume of PB used for the CellSearch system, the first and only FDA‐approved kit for epithelial‐origin CTCs enumeration, is 7.5 ml which is a large amount of blood for children. These obstacles could be solved by identifying NB‐specific cell‐surface antigen in NB through a high‐accuracy sequencing platform, e.g., single‐cell RNA or DNA sequencing. More standardized effective enrichment and collection protocols for NB CTCs are needed to avoid a large sample volume for children and ensure reliable results in routine clinical use.

## AUTHOR CONTRIBUTIONS

Ran Yang collected references and wrote the manuscript. Shan Zheng and Rui Dong critically revised and edited the manuscript. All authors read and approved the final manuscript.

## CONFLICT OF INTEREST

The authors declare no conflicts of interest concerning the materials used in this paper.

## ETHICS STATEMENT

Not applicable.

## Data Availability

All information is provided in the manuscript.

## References

[1] Stiller CA , Parkin DM . International variations in the incidence of neuroblastoma. Int J Cancer. 1992;52(4):538‐543. 1399133 10.1002/ijc.2910520407

[2] Tsubota S , Kadomatsu K . Origin and initiation mechanisms of neuroblastoma. Cell Tissue Res. 2018;372(2):211‐221. 29445860 10.1007/s00441-018-2796-z

[3] Cohn SL , Pearson AD , London WB , et al. The International Neuroblastoma Risk Group (INRG) classification system: an INRG Task Force report. J Clin Oncol. 2009;27(2):289‐297. 19047291 10.1200/JCO.2008.16.6785 PMC2650388

[4] Cangemi G , Reggiardo G , Barco S , et al. Prognostic value of ferritin, neuron‐specific enolase, lactate dehydrogenase, and urinary and plasmatic catecholamine metabolites in children with neuroblastoma. Onco Targets Ther. 2012;5:417‐423. 23226699 10.2147/OTT.S36366 PMC3514851

[5] Heitzer E , Haque IS , Roberts CES , Speicher MR . Current and future perspectives of liquid biopsies in genomics‐driven oncology. Nat Rev Genet. 2019;20(2):71‐88. 30410101 10.1038/s41576-018-0071-5

[6] Alix‐Panabières C , Pantel K . Clinical applications of circulating tumor cells and circulating tumor DNA as liquid biopsy. Cancer Discov. 2016;6(5):479‐491. 26969689 10.1158/2159-8290.CD-15-1483

[7] Siravegna G , Marsoni S , Siena S , Bardelli A . Integrating liquid biopsies into the management of cancer. Nat Rev Clin Oncol. 2017;14(9):531‐548. 28252003 10.1038/nrclinonc.2017.14

[8] Corcoran RB , Chabner BA . Application of cell‐free DNA analysis to cancer treatment. N Engl J Med. 2018;379(18):1754‐1765. 30380390 10.1056/NEJMra1706174

[9] Pantel K , Alix‐Panabieres C . Liquid biopsy and minimal residual disease—latest advances and implications for cure. Nat Rev Clin Oncol. 2019;16(7):409‐424. 30796368 10.1038/s41571-019-0187-3

[10] Liu X , Zhang Z , Zhang B , et al. Circulating tumor cells detection in neuroblastoma patients by EpCAM‐independent enrichment and immunostaining‐fluorescence in situ hybridization. EBioMedicine. 2018;35:244‐250. 30104180 10.1016/j.ebiom.2018.08.005 PMC6154868

[11] Ashworth T . A case of cancer in which cells similar to those in the tumours were seen in the blood after death. Aust Med J. 1869;14:146.

[12] Harney AS , Arwert EN , Entenberg D , et al. Real‐time imaging reveals local, transient vascular permeability, and tumor cell intravasation stimulated by TIE2hi macrophage‐derived VEGFA. Cancer Discov. 2015;5(9):932‐943. 26269515 10.1158/2159-8290.CD-15-0012 PMC4560669

[13] Keller L , Pantel K . Unravelling tumour heterogeneity by single‐cell profiling of circulating tumour cells. Nat Rev Cancer. 2019;19(10):553‐567. 31455893 10.1038/s41568-019-0180-2

[14] Weis SM , Cheresh DA . Tumor angiogenesis: molecular pathways and therapeutic targets. Nat Med. 2011;17(11):1359‐1370. 22064426 10.1038/nm.2537

[15] Mohme M , Riethdorf S , Pantel K . Circulating and disseminated tumour cells—mechanisms of immune surveillance and escape. Nat Rev Clin Oncol. 2017;14(3):155‐167. 27644321 10.1038/nrclinonc.2016.144

[16] Dasgupta A , Lim AR , Ghajar CM . Circulating and disseminated tumor cells: harbingers or initiators of metastasis? Mol Oncol. 2017;11(1):40‐61. 28085223 10.1002/1878-0261.12022 PMC5423226

[17] Te Boekhorst V , Friedl P . Plasticity of cancer cell invasion‐mechanisms and implications for therapy. Adv Cancer Res. 2016;132:209‐264. 27613134 10.1016/bs.acr.2016.07.005

[18] Georgouli M , Herraiz C , Crosas‐Molist E , et al. Regional activation of myosin II in cancer cells drives tumor progression via a secretory cross‐talk with the immune microenvironment. Cell. 2019;176(4):757‐74.e23. 30712866 10.1016/j.cell.2018.12.038 PMC6370915

[19] Gkountela S , Castro‐Giner F , Szczerba BM , et al. Circulating tumor cell clustering shapes DNA methylation to enable metastasis seeding. Cell. 2019;176(1–2):98‐112.e14. 30633912 10.1016/j.cell.2018.11.046 PMC6363966

[20] Aceto N , Bardia A , Miyamoto DT , et al. Circulating tumor cell clusters are oligoclonal precursors of breast cancer metastasis. Cell. 2014;158(5):1110‐1122. 25171411 10.1016/j.cell.2014.07.013 PMC4149753

[21] Kozminsky M , Fouladdel S , Chung JS , et al. Detection of CTC clusters and a dedifferentiated RNA‐expression survival signature in prostate cancer. Adv Sci. 2019;6(2):1801254. 10.1002/advs.201801254 PMC6343066 30693182

[22] Schuster E , Taftaf R , Reduzzi C , Albert MK , Romero‐Calvo I , Liu H . Better together: circulating tumor cell clustering in metastatic cancer. Trends in Cancer. 2021;7(11):1020‐1032. 34481763 10.1016/j.trecan.2021.07.001 PMC8541931

[23] Szczerba BM , Castro‐Giner F , Vetter M , et al. Neutrophils escort circulating tumour cells to enable cell cycle progression. Nature. 2019;566(7745):553‐557. 30728496 10.1038/s41586-019-0915-y

[24] Ao Z , Shah SH , Machlin LM , et al. Identification of cancer‐associated fibroblasts in circulating blood from patients with metastatic breast cancer. Cancer Res. 2015;75(22):4681‐4687. 26471358 10.1158/0008-5472.CAN-15-1633

[25] Jones ML , Siddiqui J , Pienta KJ , Getzenberg RH . Circulating fibroblast‐like cells in men with metastatic prostate cancer. Prostate. 2013;73(2):176‐181. 22718300 10.1002/pros.22553 PMC3482413

[26] Richardson AM , Havel LS , Koyen AE , et al. Vimentin is required for lung adenocarcinoma metastasis via heterotypic tumor cell–cancer‐associated fibroblast interactions during collective invasion. Clin Cancer Res. 2018;24(2):420‐432. 29208669 10.1158/1078-0432.CCR-17-1776 PMC5771825

[27] Haber DA , Velculescu VE . Blood‐based analyses of cancer: circulating tumor cells and circulating tumor DNA. Cancer Discov. 2014;4(6):650‐661. 24801577 10.1158/2159-8290.CD-13-1014 PMC4433544

[28] Alix‐Panabières C , Pantel K . Challenges in circulating tumour cell research. Nat Rev Cancer. 2014;14(9):623‐631. 25154812 10.1038/nrc3820

[29] Meng S , Tripathy D , Frenkel EP , et al. Circulating tumor cells in patients with breast cancer dormancy. Clin Cancer Res. 2004;10(24):8152‐8162. 15623589 10.1158/1078-0432.CCR-04-1110

[30] Gerson JM , Schlesinger HR , Sereni P , Moorhead PS , Hummeler K . Isolation and characterization of a neuroblastoma cell line from peripheral blood in a patient with disseminated disease. Cancer. 1977;39(6):2508‐2512. 17466 10.1002/1097-0142(197706)39:6<2508::aid-cncr2820390630>3.0.co;2-x

[31] Moss TJ , Cairo M , Santana VM , Weinthal J , Hurvitz C , Bostrom B . Clonogenicity of circulating neuroblastoma cells: implications regarding peripheral blood stem cell transplantation. Blood. 1994;83(10):3085‐3089. 7910052

[32] Kreissman SG , Seeger RC , Matthay KK , et al. Purged versus non‐purged peripheral blood stem‐cell transplantation for high‐risk neuroblastoma (COG A3973): a randomised phase 3 trial. Lancet Oncol. 2013;14(10):999‐1008. 23890779 10.1016/S1470-2045(13)70309-7 PMC3963485

[33] Seeger RC , Reynolds CP , Gallego R , Stram DO , Gerbing RB , Matthay KK . Quantitative tumor cell content of bone marrow and blood as a predictor of outcome in stage IV neuroblastoma: a Children's Cancer Group study. J Clin Oncol. 2000;18(24):4067‐4076. 11118468 10.1200/JCO.2000.18.24.4067

[34] Miyajima Y , Horibe K , Fukuda M , et al. Sequential detection of tumor cells in the peripheral blood and bone marrow of patients with stage IV neuroblastoma by the reverse transcription‐polymerase chain reaction for tyrosine hydroxylase mRNA. Cancer. 1996;77(6):1214‐1219. 8635146 10.1002/(sici)1097-0142(19960315)77:6<1214::aid-cncr31>3.0.co;2-2

[35] Jain M , Kumar A , Mishra S , Verma N , Goel MM . Circulating tumor cells in neuroblastoma. Turk J Haematol. 2017;34(4):369‐370. 28840849 10.4274/tjh.2017.0199 PMC5774353

[36] Olm F , Panse L , Dykes JH , Bexell D , Laurell T , Scheding S . Label‐free separation of neuroblastoma patient‐derived xenograft (PDX) cells from hematopoietic progenitor cell products by acoustophoresis. Stem Cell Res Ther. 2021;12(1):542. 34654486 10.1186/s13287-021-02612-2 PMC8518319

[37] Beiske K , Burchill SA , Cheung IY , et al. Consensus criteria for sensitive detection of minimal neuroblastoma cells in bone marrow, blood and stem cell preparations by immunocytology and QRT‐PCR: recommendations by the International Neuroblastoma Risk Group Task Force. Br J Cancer. 2009;100(10):1627‐1637. 19401690 10.1038/sj.bjc.6605029 PMC2696761

[38] Batth IS , Dao L , Satelli A , et al. Cell surface vimentin positive circulating tumor cell‐based relapse prediction in a long‐term longitudinal study of post‐remission neuroblastoma patients. Int J Cancer. 2020;147:3550‐3559. 32506485 10.1002/ijc.33140 PMC7839076

[39] Kirby BJ , Jodari M , Loftus MS , et al. Functional characterization of circulating tumor cells with a prostate‐cancer‐specific microfluidic device. PLoS One. 2012;7(4):e35976. 22558290 10.1371/journal.pone.0035976 PMC3338784

[40] Ozkumur E , Shah AM , Ciciliano JC , et al. Inertial focusing for tumor antigen‐dependent and ‐independent sorting of rare circulating tumor cells. Sci Transl Med. 2013;5(179):179ra47. 10.1126/scitranslmed.3005616 PMC3760275 23552373

[41] Lanino E , Melodia A , Casalaro A , Cornaglia‐Ferraris P . Neuroblastoma cells circulate in peripheral blood. Pediatr Hematol Oncol. 1989;6(2):193‐195. 2702074 10.3109/08880018909034286

[42] Sanders DG , Wiley FM , Moss TJ . Serial immunocytologic analysis of blood for tumor cells in two patients with neuroblastoma. Cancer. 1991;67(5):1423‐1427. 1991307 10.1002/1097-0142(19910301)67:5<1423::aid-cncr2820670525>3.0.co;2-l

[43] Moss TJ , Sanders DG . Detection of neuroblastoma cells in blood. J Clin Oncol. 1990;8(4):736‐740. 2179482 10.1200/JCO.1990.8.4.736

[44] Mattano LA Jr , Moss TJ , Emerson SG . Sensitive detection of rare circulating neuroblastoma cells by the reverse transcriptase‐polymerase chain reaction. Cancer Res. 1992;52(17):4701‐4705. 1380888

[45] Lee NH , Son MH , Choi YB , et al. Clinical significance of tyrosine hydroxylase mRNA transcripts in peripheral blood at diagnosis in patients with neuroblastoma. Cancer Res Treat. 2016;48(4):1399‐1407. 27034145 10.4143/crt.2015.481 PMC5080821

[46] Yáñez Y , Hervás D , Grau E , et al. TH and DCX mRNAs in peripheral blood and bone marrow predict outcome in metastatic neuroblastoma patients. J Cancer Res Clin Oncol. 2016;142(3):573‐580. 26498952 10.1007/s00432-015-2054-7 PMC11819078

[47] Gilbert J , Norris MD , Marshall GM , Haber M . Low specificity of PGP9.5 expression for detection of micrometastatic neuroblastoma. Br J Cancer. 1997;75(12):1779‐1781. 9192981 10.1038/bjc.1997.303 PMC2223599

[48] Corrias MV , Haupt R , Carlini B , et al. Multiple target molecular monitoring of bone marrow and peripheral blood samples from patients with localized neuroblastoma and healthy donors. Pediatr Blood Cancer. 2012;58(1):43‐49. 21254375 10.1002/pbc.22960

[49] Marachelian A , Villablanca JG , Liu CW , et al. Expression of five neuroblastoma genes in bone marrow or blood of patients with relapsed/refractory neuroblastoma provides a new biomarker for disease and prognosis. Clin Cancer Res. 2017;23(18):5374‐5383. 28559462 10.1158/1078-0432.CCR-16-2647

[50] Swerts K , De Moerloose B , Dhooge C , et al. Potential application of ELAVL4 real‐time quantitative reverse transcription‐PCR for detection of disseminated neuroblastoma cells. Clin Chem. 2006;52(3):438‐445. 16384890 10.1373/clinchem.2005.059485

[51] Thwin KKM , Ishida T , Uemura S , et al. Level of seven neuroblastoma‐associated mRNAs detected by droplet digital PCR is associated with tumor relapse/regrowth of high‐risk neuroblastoma patients. J Mol Diagn. 2020;22(2):236‐246. 31837427 10.1016/j.jmoldx.2019.10.012

[52] Stutterheim J , Gerritsen A , Zappeij‐Kannegieter L , et al. PHOX2B is a novel and specific marker for minimal residual disease testing in neuroblastoma. J Clin Oncol. 2008;26(33):5443‐5449. 18838715 10.1200/JCO.2007.13.6531

[53] Stutterheim J , Gerritsen A , Zappeij‐Kannegieter L , et al. Detecting minimal residual disease in neuroblastoma: the superiority of a panel of real‐time quantitative PCR markers. Clin Chem. 2009;55(7):1316‐1326. 19460840 10.1373/clinchem.2008.117945

[54] Cheung IY , Feng Y , Gerald W , Cheung NK . Exploiting gene expression profiling to identify novel minimal residual disease markers of neuroblastoma. Clin Cancer Res. 2008;14(21):7020‐7027. 18980998 10.1158/1078-0432.CCR-08-0541 PMC2670609

[55] Viprey VF , Lastowska MA , Corrias MV , Swerts K , Jackson MS , Burchill SA . Minimal disease monitoring by QRT‐PCR: guidelines for identification and systematic validation of molecular markers prior to evaluation in prospective clinical trials. J Pathol. 2008;216(2):245‐252. 18702176 10.1002/path.2406

[56] Corrias MV , Faulkner LB , Pistorio A , et al. Detection of neuroblastoma cells in bone marrow and peripheral blood by different techniques: accuracy and relationship with clinical features of patients. Clin Cancer Res. 2004;10(23):7978‐7985. 15585633 10.1158/1078-0432.CCR-04-0815

[57] Burchill SA , Bradbury FM , Smith B , Lewis IJ , Selby P . Neuroblastoma cell detection by reverse transcriptase‐polymerase chain reaction (RT‐PCR) for tyrosine hydroxylase mRNA. Int J Cancer. 1994;57(5):671‐675. 7910809 10.1002/ijc.2910570510

[58] Merugu S , Chen L , Gavens E , et al. Detection of circulating and disseminated neuroblastoma cells using the ImageStream flow cytometer for use as predictive and pharmacodynamic biomarkers. Clin Cancer Res. 2020;26(1):122‐134. 31767563 10.1158/1078-0432.CCR-19-0656

[59] Carpenter EL , Rader J , Ruden J , et al. Dielectrophoretic capture and genetic analysis of single neuroblastoma tumor cells. Front Oncol. 2014;4:201. 25133137 10.3389/fonc.2014.00201 PMC4116800

[60] Yáñez Y , Grau E , Oltra S , et al. Minimal disease detection in peripheral blood and bone marrow from patients with non‐metastatic neuroblastoma. J Cancer Res Clin Oncol. 2011;137(8):1263‐1272. 21706131 10.1007/s00432-011-0997-x PMC11828153

[61] Viprey VF , Gregory WM , Corrias MV , et al. Neuroblastoma mRNAs predict outcome in children with stage 4 neuroblastoma: a European HR‐NBL1/SIOPEN study. J Clin Oncol. 2014;32(10):1074‐1083. 24590653 10.1200/JCO.2013.53.3604

[62] van Wezel EM , Stutterheim J , Vree F , et al. Minimal residual disease detection in autologous stem cell grafts from patients with high risk neuroblastoma. Pediatr Blood Cancer. 2015;62(8):1368‐1373. 25939774 10.1002/pbc.25507

[63] Avigad S , Feinberg‐Gorenshtein G , Luria D , et al. Minimal residual disease in peripheral blood stem cell harvests from high‐risk neuroblastoma patients. J Pediatr Hematol Oncol. 2009;31(1):22‐26. 19125082 10.1097/MPH.0b013e31818e532c

[64] Chambon F , Tchirkov A , Pereira B , Rochette E , Deméocq F , Kanold J . Molecular assessment of minimal residual disease in PBSC harvests provides prognostic information in neuroblastoma. Pediatr Blood Cancer. 2013;60(9):E109‐E112. 23596146 10.1002/pbc.24538

[65] Rill DR , Santana VM , Roberts WM , et al. Direct demonstration that autologous bone marrow transplantation for solid tumors can return a multiplicity of tumorigenic cells. Blood. 1994;84(2):380‐383. 8025266

[66] Corrias MV , Haupt R , Carlini B , et al. Peripheral blood stem cell tumor cell contamination and survival of neuroblastoma patients. Clin Cancer Res. 2006;12(19):5680‐5685. 17020970 10.1158/1078-0432.CCR-06-0740

[67] Viprey VF , Corrias MV , Kagedal B , et al. Standardisation of operating procedures for the detection of minimal disease by QRT‐PCR in children with neuroblastoma: quality assurance on behalf of SIOPEN‐R‐NET. Eur J Cancer. 2007;43(2):341‐350. 17023157 10.1016/j.ejca.2006.08.007

[68] Cheung IY , Cheung NK . Quantitation of marrow disease in neuroblastoma by real‐time reverse transcription‐PCR. Clin Cancer Res. 2001;7(6):1698‐1705. 11410509

[69] Druy AE , Shorikov EV , Tsaur GA , et al. Prospective investigation of applicability and the prognostic significance of bone marrow involvement in patients with neuroblastoma detected by quantitative reverse transcription PCR. Pediatr Blood Cancer. 2018;65(11):e27354. 30007008 10.1002/pbc.27354

[70] Klega K , Imamovic‐Tuco A , Ha G , et al. Detection of somatic structural variants enables quantification and characterization of circulating tumor DNA in children with solid tumors. JCO precis. Oncologia. 2018;2018:1‐13. 10.1200/PO.17.00285 PMC6049092 30027144

[71] Chicard M , Colmet‐Daage L , Clement N , et al. Whole‐exome sequencing of cell‐free DNA reveals Temporo‐spatial heterogeneity and identifies treatment‐resistant clones in neuroblastoma. Clin Cancer Res. 2018;24(4):939‐949. 29191970 10.1158/1078-0432.CCR-17-1586

[72] Wang X , Wang L , Su Y , et al. Plasma cell‐free DNA quantification is highly correlated to tumor burden in children with neuroblastoma. Cancer Med. 2018;7(7):3022‐3030. 29905010 10.1002/cam4.1586 PMC6051223

[73] Kurihara S , Ueda Y , Onitake Y , et al. Circulating free DNA as non‐invasive diagnostic biomarker for childhood solid tumors. J Pediatr Surg. 2015;50(12):2094‐2097. 26388126 10.1016/j.jpedsurg.2015.08.033

[74] van Zogchel LMJ , van Wezel EM , van Wijk J , et al. Hypermethylated RASSF1A as circulating tumor DNA marker for disease monitoring in neuroblastoma. JCO precis. Oncologia. 2020;4:291‐306. 10.1200/PO.19.00261 PMC7446415 32923888

[75] Lodrini M , Graef J , Thole‐Kliesch TM , et al. Targeted analysis of cell‐free circulating tumor DNA is suitable for early relapse and actionable target detection in patients with neuroblastoma. Clin Cancer Res. 2022;28(9):1809‐1820. 35247920 10.1158/1078-0432.CCR-21-3716

[76] Campbell KM , Klega KS , Shulman DS , et al. Changes in ctDNA levels after MIBG therapy in patients with relapsed or refractory neuroblastoma. J Clin Oncol. 2021;39(15 suppl):10012.

[77] Kojima M , Hiyama E , Fukuba I , et al. Detection of MYCN amplification using blood plasma: noninvasive therapy evaluation and prediction of prognosis in neuroblastoma. Pediatr Surg Int. 2013;29(11):1139‐1145. 24022278 10.1007/s00383-013-3374-9

[78] Kahana‐Edwin S , Cain LE , McCowage G , et al. Neuroblastoma molecular risk‐stratification of DNA copy number and ALK genotyping via cell‐free circulating tumor DNA profiling. Cancers. 2021;13(13):3365. 34282791 10.3390/cancers13133365 PMC8267662

[79] Combaret V , Audoynaud C , Iacono I , et al. Circulating MYCN DNA as a tumor‐specific marker in neuroblastoma patients. Cancer Res. 2002;62(13):3646‐3648. 12097268

[80] Combaret V , Iacono I , Bellini A , et al. Detection of tumor ALK status in neuroblastoma patients using peripheral blood. Cancer Med. 2015;4(4):540‐550. 25653133 10.1002/cam4.414 PMC4402069

[81] Yagyu S , Iehara T , Gotoh T , et al. Preoperative analysis of 11q loss using circulating tumor‐released DNA in serum: a novel diagnostic tool for therapy stratification of neuroblastoma. Cancer Lett. 2011;309(2):185‐189. 21726937 10.1016/j.canlet.2011.05.032

[82] Combaret V , Bréjon S , Iacono I , et al. Determination of 17q gain in patients with neuroblastoma by analysis of circulating DNA. Pediatr Blood Cancer. 2011;56(5):757‐761. 21370407 10.1002/pbc.22816

[83] Applebaum MA , Barr EK , Karpus J , et al. 5‐Hydroxymethylcytosine profiles in circulating cell‐free DNA associate with disease burden in children with neuroblastoma. Clin Cancer Res. 2020;26(6):1309‐1317. 31852832 10.1158/1078-0432.CCR-19-2829 PMC7073281

[84] Yagyu S , Gotoh T , Iehara T , et al. Circulating methylated‐DCR2 gene in serum as an indicator of prognosis and therapeutic efficacy in patients with MYCN nonamplified neuroblastoma. Clin Cancer Res. 2008;14(21):7011‐7019. 18980997 10.1158/1078-0432.CCR-08-1249

[85] Misawa A , Tanaka S , Yagyu S , et al. RASSF1A hypermethylation in pretreatment serum DNA of neuroblastoma patients: a prognostic marker. Br J Cancer. 2009;100(2):399‐404. 19165202 10.1038/sj.bjc.6604887 PMC2634715

[86] Van Roy N , Van Der Linden M , Menten B , et al. Shallow whole genome sequencing on circulating cell‐free DNA allows reliable noninvasive copy‐number profiling in neuroblastoma patients. Clin Cancer Res. 2017;23(20):6305‐6314. 28710315 10.1158/1078-0432.CCR-17-0675

[87] Duan C , Wang H , Chen Y , et al. Whole exome sequencing reveals novel somatic alterations in neuroblastoma patients with chemotherapy. Cancer Cell Int. 2018;18(1):21. 29467591 10.1186/s12935-018-0521-3 PMC5816515

[88] Lodrini M , Wünschel J , Thole‐Kliesch TM , et al. Circulating cell‐free DNA assessment in biofluids from children with neuroblastoma demonstrates feasibility and potential for minimally invasive molecular diagnostics. Cancer. 2022;14(9):2080. 10.3390/cancers14092080 PMC9099910 35565208

[89] Beltran H , Jendrisak A , Landers M , et al. The initial detection and partial characterization of circulating tumor cells in neuroendocrine prostate cancer. Clin Cancer Res. 2016;22(6):1510‐1519. 26671992 10.1158/1078-0432.CCR-15-0137 PMC4990782

[90] Shaw JA , Guttery DS , Hills A , et al. Mutation analysis of cell‐free DNA and single circulating tumor cells in metastatic breast cancer patients with high circulating tumor cell counts. Clin Cancer Res. 2017;23(1):88‐96. 27334837 10.1158/1078-0432.CCR-16-0825 PMC6241844

[91] Rifatbegovic F , Frech C , Abbasi MR , et al. Neuroblastoma cells undergo transcriptomic alterations upon dissemination into the bone marrow and subsequent tumor progression. Int J Cancer. 2018;142(2):297‐307. 28921546 10.1002/ijc.31053 PMC5725737

[92] Gao Y , Ni X , Guo H , et al. Single‐cell sequencing deciphers a convergent evolution of copy number alterations from primary to circulating tumor cells. Genome Res. 2017;27(8):1312‐1322. 28487279 10.1101/gr.216788.116 PMC5538548

[93] Grobner SN , Worst BC , Weischenfeldt J , et al. The landscape of genomic alterations across childhood cancers. Nature. 2018;555(7696):321‐327. 29489754 10.1038/nature25480

[94] Ledergor G , Weiner A , Zada M , et al. Single cell dissection of plasma cell heterogeneity in symptomatic and asymptomatic myeloma. Nat Med. 2018;24(12):1867‐1876. 30523328 10.1038/s41591-018-0269-2

[95] Reza KK , Dey S , Wuethrich A , et al. In situ single cell proteomics reveals circulating tumor cell heterogeneity during treatment. ACS Nano. 2021;15:11231‐11243. 34225455 10.1021/acsnano.0c10008

[96] Donato C , Buczak K , Schmidt A , Aceto N . Mass spectrometry analysis of circulating breast cancer cells from a xenograft mouse model. STAR Protoc. 2021;2(2):100480. 33982014 10.1016/j.xpro.2021.100480 PMC8082161

[97] Sinkala E , Sollier‐Christen E , Renier C , et al. Profiling protein expression in circulating tumour cells using microfluidic western blotting. Nat Commun. 2017;8(1):14622. 28332571 10.1038/ncomms14622 PMC5376644

[98] Yu M , Bardia A , Aceto N , et al. Cancer therapy. Ex vivo culture of circulating breast tumor cells for individualized testing of drug susceptibility. Science. 2014;345(6193):216‐220. 25013076 10.1126/science.1253533 PMC4358808

[99] Cayrefourcq L , Mazard T , Joosse S , et al. Establishment and characterization of a cell line from human circulating colon cancer cells. Cancer Res. 2015;75(5):892‐901. 25592149 10.1158/0008-5472.CAN-14-2613

[100] Baccelli I , Schneeweiss A , Riethdorf S , et al. Identification of a population of blood circulating tumor cells from breast cancer patients that initiates metastasis in a xenograft assay. Nat Biotechnol. 2013;31(6):539‐544. 23609047 10.1038/nbt.2576

[101] Stewart CA , Gay CM , Xi Y , et al. Single‐cell analyses reveal increased intratumoral heterogeneity after the onset of therapy resistance in small‐cell lung cancer. Nat Cancer. 2020;1(4):423‐436. 33521652 10.1038/s43018-019-0020-z PMC7842382

[102] Cooper MA , Fehniger TA , Caligiuri MA . The biology of human natural killer‐cell subsets. Trends Immunol. 2001;22(11):633‐640. 11698225 10.1016/s1471-4906(01)02060-9

[103] Yagyu S , Iehara T , Tanaka S , et al. Serum‐based quantification of MYCN gene amplification in Young patients with neuroblastoma: potential utility as a surrogate biomarker for neuroblastoma. PLoS One. 2016;11(8):e0161039. 27513929 10.1371/journal.pone.0161039 PMC4981470

[104] Combaret V , Bergeron C , Noguera R , Iacono I , Puisieux A . Circulating MYCN DNA predicts MYCN‐amplification in neuroblastoma. J Clin Oncol. 2005;23(34):8919‐8920. author reply 20. 16314658 10.1200/JCO.2005.04.0170

[105] Chicard M , Boyault S , Colmet Daage L , et al. Genomic copy number profiling using circulating free tumor DNA highlights heterogeneity in neuroblastoma. Clin Cancer Res. 2016;22(22):5564‐5573. 27440268 10.1158/1078-0432.CCR-16-0500

[106] Yang Q , Kiernan CM , Tian Y , et al. Methylation of CASP8, DCR2, and HIN‐1 in neuroblastoma is associated with poor outcome. Clin Cancer Res. 2007;13(11):3191‐3197. 17545522 10.1158/1078-0432.CCR-06-2846

[107] Cimmino F , Lasorsa VA , Vetrella S , Iolascon A , Capasso M . A targeted gene panel for circulating tumor DNA sequencing in neuroblastoma. Front Oncol. 2020;10:596191. 33381456 10.3389/fonc.2020.596191 PMC7769379

